# Maternal late-pregnancy BMI is associated with compartment-specific immune-metabolic signatures across the maternal–placental–fetal axis

**DOI:** 10.3389/fcell.2026.1899628

**Published:** 2026-10-28

**Authors:** Viviane Schuch, Alaijah Bashi, Daniel Hossack, Toidi Adekambi, Tyana Joseph, Cristina Wilson, Raimot Olanrewaju, Amber Dawning, Ariyana Johnson, Skye Jones, Elise Sintim-Aboagye, Elizabeth Ann L. Enninga, Rana Chakraborty, Erica L. Johnson

**Affiliations:** 1 Department of Microbiology, Biochemistry, and Immunology, Morehouse School of Medicine, Atlanta, GA, United States; 2 Department of Obstetrics and Gynecology, Morehouse School of Medicine, Atlanta, GA, United States; 3 Department of Obstetrics and Gynecology, Mayo Clinic, Rochester, MN, United States; 4 Department of Pediatrics, Division of Pediatric Infectious Disease, Miller School of Medicine, University of Miami, Miami, FL, United States

**Keywords:** CBMC, maternal–placental–fetal axis, PBMC, placenta, third-trimester maternal BMI

## Abstract

**Introduction:**

Elevated maternal body mass index (BMI) during pregnancy is associated with adverse maternal and offspring outcomes; yet the molecular pathways linking maternal metabolic status to placental function and fetal immune development remain poorly understood.

**Methods:**

We performed multi-compartment transcriptomic profiling across the maternal–placental–fetal axis, complemented by exploratory cytokine profiling in a larger available plasma sample set. Maternal peripheral blood mononuclear cells (PBMCs), placental villous tissue, cord blood mononuclear cells (CBMCs), maternal plasma, and cord plasma were collected at delivery when available. RNA-seq data were available for 17 maternal PBMC samples, 18 CBMC samples, and 14 placental samples from 20 term pregnancies, while Luminex profiling was performed in 31 maternal plasma and 31 cord plasma samples, comprising available plasma from the RNA-seq cohort plus additional plasma-only samples. RNA-seq analyses were performed separately within each compartment using third-trimester BMI as a continuous variable, followed by Hallmark pathway enrichment, co-expression network analysis, cytokine/chemokine profiling and targeted placental qRT-PCR validation.

**Results:**

Higher maternal third-trimester BMI was associated with distinct transcriptional programs across maternal, placental, and fetal compartments. In maternal PBMCs, higher third-trimester BMI was associated with a modest but coordinated gene-level signature and pathway patterns consistent with selective immune activation, alongside broader attenuation of metabolic, inflammatory, and redox-related programs. Placental tissue exhibited the strongest BMI-associated transcriptomic signature, characterized by metabolic and stress-response activation, including enrichment of oxidative phosphorylation, reactive oxygen species signaling, unfolded protein response, DNA repair, MTORC1 signaling, and adipogenesis. Exploratory co-expression analyses further highlighted candidate extracellular matrix, vascular-interface, and placental endocrine-associated modules. In CBMCs, higher third-trimester BMI was associated with fetal immune-developmental remodeling, including proliferative pathway enrichment and relative attenuation of inflammatory and immune-response signaling. Exploratory plasma cytokine profiling identified nominal compartment-specific associations with higher third-trimester BMI; however, none remained significant after multiple-testing correction.

**Discussion:**

Collectively, the multi-compartment findings nominate candidate mitochondrial, inflammatory, vascular-interface, extracellular matrix, and placental endocrine-associated pathways for validation in larger independent cohorts.

## Graphical Abstract

**Figure:**
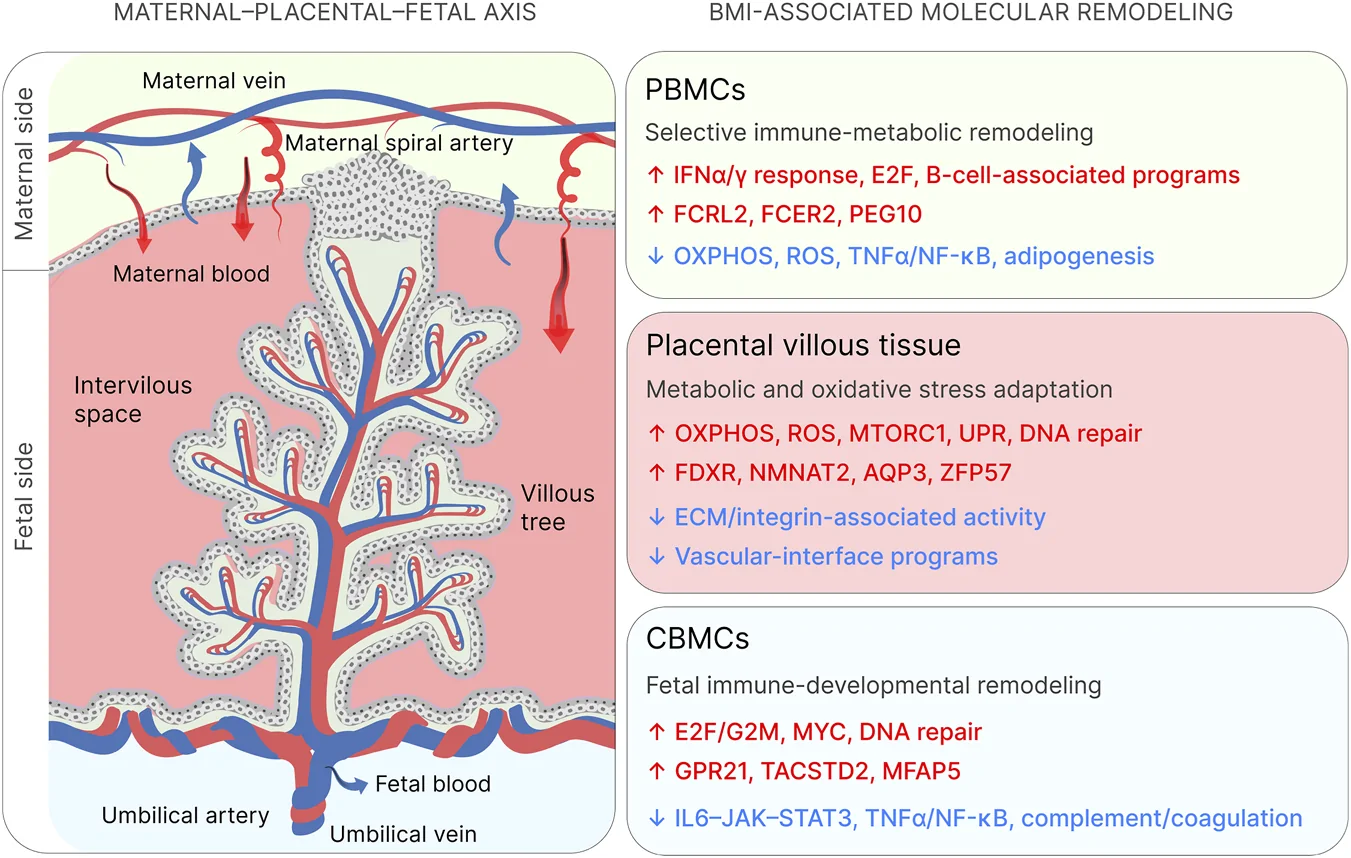
Overview of BMI-associated molecular remodeling across the maternal–placental–fetal axis. Schematic summary of the study design and major findings, highlighting compartment-specific immune-metabolic remodeling associated with increasing third-trimester BMI across maternal PBMCs, placental tissue, CBMCs, and plasma immune mediators. Adapted from Schuch et al. (2026), under the CC BY license.

## 1 Introduction

The prevalence of obesity, defined in adults as a body mass index (BMI) ≥30 kg/m^2^, has increased worldwide over the last 60 years and represents a major public health concern, particularly in women of reproductive age (Ng et al., 2014; Catalano and Shankar, 2017). During pregnancy, pre-pregnancy obesity and elevated maternal BMI are associated with adverse maternal and neonatal outcomes, including gestational diabetes, preeclampsia, indicated preterm birth, fetal macrosomia, fetal growth restriction, stillbirth, and increased risk of congenital anomalies such as neural tube defects (Ramachenderan et al., 2008; Leddy et al., 2008; Fitzsimons et al., 2009). Offspring born to mothers with obesity also have increased risk of obesity, metabolic disease, neuropsychiatric disorders, cognitive impairment, and dysregula infted immune responses in postnatal life (Howell and Powell, 2017; Zhang et al., 2018; Martí et al., 2001). These associations suggest that maternal metabolic status plays an important role in regulating placental function and fetal development; however, the molecular pathways linking elevated maternal BMI to maternal–fetal outcomes are poorly characterized.

Pregnancy requires coordinated immune, metabolic, endocrine, and vascular adaptations that support fetal growth while maintaining maternal–fetal tolerance (Burton and Fowden, 2015). The placenta is central to this process, serving as a transient but highly specialized interface that regulates nutrient transport, hormone production, immune communication, and maternal–fetal exchange (Burton and Fowden, 2015; Napso et al., 2018). In pregnancies complicated by obesity or elevated maternal BMI, the intrauterine environment is characterized by metabolic dysfunction and chronic low-grade inflammation, which can disrupt placental development, maternal–fetal immune homeostasis, and fetal growth (Kislal et al., 2020; Monaco-Brown and Lawrence, 2022). Maternal obesity is associated with altered circulating metabolites, adipokines, growth factors, and inflammatory mediators, including glucose, lipids, leptin, adiponectin, insulin-like growth factors, IL-6, IL-8, TNF-α, MCP-1, and C-reactive protein (Kelly et al., 2020; Ritter et al., 2020; Louwen et al., 2018). These maternal signals can influence placental function directly through altered nutrient transport, inflammatory activation, trophoblast differentiation, vascular remodeling, and oxidative-stress responses (Kelly et al., 2020; Vaughan et al., 2017; Jansson and Powell, 2013; Aye et al., 2014; Myatt and Maloyan, 2016; Migda et al., 2016; Santos-Rosendo et al., 2020; Dimasuay et al., 2016).

The placenta is also considered a key mediator of developmental programming because it integrates maternal metabolic and inflammatory signals and shapes fetal exposure to nutrients, hormones, cytokines, and oxygen availability (Jansson and Powell, 2013; Dimasuay et al., 2016; Barker and Thornburg, 2013). Epidemiological and experimental studies suggest that maternal obesity and placental dysfunction are linked to long-term offspring risk of cardiometabolic, immune, respiratory, and neurodevelopmental disorders (LaCoursiere et al., 2010; Nguyen et al., 2015; Rivera et al., 2015; Stang and Huffman, 2016; Barker and Thornburg, 2013; Thornburg, 2011; Eriksson et al., 2011; Zhang et al., 2011; Armitage et al., 2004; Armitage et al., 2005; Ford et al., 2009; Bolton and Bilbo, 2014; Adane et al., 2016; Hinkle et al., 2013; Tanda et al., 2013; Beneventi et al., 2023; Leibowitz et al., 2012; Forno et al., 2014; O’Reilly and Reynolds, 2013). However, most studies have focused on single compartments, such as maternal blood, placenta, or cord blood, making it difficult to determine how maternal metabolic status is reflected *in tandem* across the maternal–placental–fetal axis. A multi-compartment approach is therefore required to distinguish systemic maternal immune changes, placental adaptations, and fetal immune-developmental signatures associated with maternal BMI.

In this study, we investigated BMI-associated molecular remodeling across maternal blood, placental tissue, and fetal cord blood samples collected from the same pregnancy cohort. We hypothesized that increasing maternal third-trimester BMI would be associated with coordinated but compartment-specific transcriptomic and immune-mediated changes across the maternal–placental–fetal axis. To test our hypothesis, we performed transcriptomic profiling of maternal peripheral blood mononuclear cells, placental villous tissue, and cord blood mononuclear cells, together with multiplex cytokine and chemokine profiling of maternal and cord plasma. By integrating gene-level differential expression, Hallmark pathway enrichment, co-expression network analysis, plasma cytokine profiling and targeted placental qRT-PCR, we aimed to identify exploratory maternal, placental, and fetal molecular signatures associated with maternal third-trimester BMI and define candidate pathways linking maternal metabolic status to placental biology and fetal immune development.

## 2 Materials and methods

### 2.1 Ethics statement/study cohort

This study was approved by the Morehouse School of Medicine Institutional Review Board (1964407-8) and the Grady Research Oversight Committee. Written informed consent was obtained from all participants. Maternal peripheral blood, umbilical cord blood, and term placental tissue (>37 weeks of gestation) were collected from hepatitis B- and HIV-1-seronegative pregnant participants (>18 years of age) immediately after elective cesarean section without labor at Grady Memorial Hospital (Atlanta, GA) between June 2023 and April 2025. Samples were transported to the laboratory within 1 h of collection. Demographic, anthropometric, clinical, and neonatal data were abstracted from the electronic medical record (EHR) and managed in REDCap. These data included third-trimester BMI, race and ethnicity, blood pressure, blood glucose, pregnancy complications, infant sex, birth weight, birth length, and other medical conditions. The primary exposure was maternal BMI recorded during the third trimester. Pre-pregnancy and early-pregnancy BMI were not used due to incomplete data (pre-pregnancy BMI was calculable for only 11 of 20 RNA-seq participants [55%]). Participant-level contributions to each analysis are summarized in Supplementary Figure S1A.

### 2.2 Isolation of peripheral and cord blood mononuclear cells

Maternal peripheral blood and umbilical cord blood were collected into sodium citrate vacutainers (BD Biosciences, Franklin Lakes, NJ, United States) immediately before or after delivery, respectively. Plasma was separated, aliquoted, and stored at −80 °C until cytokine and chemokine analysis. Maternal peripheral blood mononuclear cells (PBMCs) and cord blood mononuclear cells (CBMCs) were isolated by Ficoll density-gradient centrifugation using a standard mononuclear cell isolation protocol. Briefly, anticoagulated blood was diluted with sterile buffer, layered over Ficoll density medium, and centrifuged to separate the mononuclear cell layer. PBMCs and CBMCs were collected from the interface, washed, resuspended in freezing medium containing fetal bovine serum and 10% dimethyl sulfoxide (DMSO), and stored in liquid nitrogen until downstream processing.

### 2.3 Placental collection and tissue processing

Membrane-free villous placental tissue was collected immediately after delivery and processed as previously described (Svensson-Arvelund et al., 2015). Briefly, placental tissue was thoroughly washed in Hank’s balanced salt solution (HBSS) to minimize peripheral blood contamination and mechanically minced. Tissue was digested in complete medium containing 0.2% trypsin/EDTA (Sigma-Aldrich, St. Louis, MO, United States) for 1 h, followed by digestion with collagenase A (1 mg/mL; Worthington Biochemical, Lakewood, NJ, United States) and DNase I (0.2 mg/mL; Sigma-Aldrich) for 1 h at 37 °C in a shaking water bath. The digested tissue was washed with PBS and passed through gauze and a 70-μm cell strainer (BD Falcon, Lexington, TN, United States). The processed villous placental tissue preparation was stored for downstream RNA extraction.

### 2.4 RNA-sequencing and preprocessing

Total RNA was extracted from PBMCs, CBMCs, and placental samples using the RNeasy kit (Qiagen), according to the manufacturer’s instructions. RNA quantity and quality were assessed using NanoDrop and Bioanalyzer instruments, and samples with RNA integrity number (RIN) > 7 were retained for sequencing. Libraries were prepared and sequenced by BGI using 150-bp paired-end sequencing, targeting ≥30 million reads per sample. Reads were quality-filtered using SOAPnuke (Chen et al., 2018) and aligned to the human reference genome GRCh38 using HISAT2 (Kim et al., 2015). Gene-level expected counts and transcripts per million (TPM) values were quantified using RSEM (Li and Dewey, 2011). Lowly expressed genes were removed by retaining genes with counts per million (CPM) > 1 in at least two samples. For exploratory PCA and MDS analyses, lowly expressed genes were filtered by retaining genes with CPM >1 in at least two samples, followed by TMM normalization and voom transformation. For DESeq2 differential expression analyses, unnormalized gene-level counts were used with compartment-specific filtering as described below (Robinson and Oshlack, 2010; Law et al., 2014; Ritchie et al., 2015).

### 2.5 Differential expression analysis

Differential expression analysis was performed separately for PBMCs, CBMCs, and placental tissue using DESeq2 (Love et al., 2014). For each compartment, samples with non-missing maternal third-trimester BMI were retained, and genes with total counts <10 were removed. Third-trimester BMI was standardized within each compartment and modeled as a continuous predictor. Batch was included as a covariate when more than one batch level was present, resulting in a full model of ∼ Batch + BMI_scaled; otherwise, the reduced model ∼ BMI_scaled was used. Wald tests were used to estimate the coefficient for standardized BMI. To improve interpretability, BMI-associated effect sizes were also rescaled to represent the expected expression change per 5-unit increase in BMI by multiplying the standardized BMI coefficient by 5/SD (BMI) within each compartment. Genes with Benjamini–Hochberg-adjusted P values < 0.10 were considered differentially expressed in compartment-specific exploratory analyses (Benjamini and Hochberg, 1995).

### 2.6 Gene-set enrichment and pathway annotation

Hallmark pathway enrichment was performed separately for each tissue using fgseaMultilevel (Korotkevich et al., 2021). Genes were ranked by the DESeq2 Wald statistic for the BMI coefficient after removing genes with missing statistics. Hallmark gene sets for *Homo sapiens* were obtained from MSigDB (Liberzon et al., 2015). Enrichment analysis was performed with gene-set sizes constrained to 10–500 genes. Pathways with Benjamini–Hochberg-adjusted P values < 0.05 were considered significantly enriched. Positive normalized enrichment scores indicated pathways enriched with increasing third-trimester BMI, whereas negative normalized enrichment scores indicated pathways depleted with increasing third-trimester BMI.

### 2.7 Co-expression network analysis

Co-expression analysis was performed separately for PBMCs, CBMCs, and placental tissue using CEMiTool (Russo et al., 2018). For each compartment, samples with non-missing maternal third-trimester BMI were retained, genes with total counts <10 were removed, and counts were variance-stabilized using DESeq2::vst with a null design (∼1) (Love et al., 2014). When applicable, batch-associated variation was adjusted using limma::removeBatchEffect while preserving biological variables in the design matrix for unsupervised network discovery (Ritchie et al., 2015). CEMiTool was then run on each compartment-specific expression matrix with internal filtering enabled and automatic soft-threshold selection. Module membership and module eigengenes were extracted from each CEMiTool object for downstream association testing and visualization. For visualization, compartment-specific third-trimester BMI tertiles were generated and used to summarize module activity across the BMI distribution. Because of the modest compartment-specific sample sizes, CEMiTool analyses were considered exploratory and were used to identify coordinated gene-expression programs that supported interpretation of the differential expression and pathway-enrichment results, rather than as an independent confirmatory discovery layer.

### 2.8 Module association, enrichment, and interaction overlays

Associations between module eigengenes and maternal third-trimester BMI were tested within each compartment using linear regression with third-trimester BMI as a continuous predictor. Covariates such as batch or sex were included only when they had not already been regressed out upstream and were estimable within the compartment-specific dataset. P values were adjusted within each compartment using the Benjamini–Hochberg procedure (Benjamini and Hochberg, 1995). Functional interpretation of co-expression modules was performed by pathway over-representation analysis using Reactome (Milacic et al., 2024) through the CEMiTool workflow. For selected modules, protein–protein interaction overlays were added using a user-supplied two-column gene-symbol interaction network compatible with CEMiTool, and module-specific interaction graphs were visualized for representative modules in each compartment. STRING-supported PPI subnetworks were used only as external annotation overlays to aid biological interpretation of selected modules; they should not be interpreted as direct evidence of physical interactions or regulatory relationships occurring in the study samples.

### 2.9 Detection and analysis of plasma cytokines/chemokines

A panel of 71 plasma cytokines and chemokines was quantified in duplicate in maternal and cord plasma using the Human Cytokine/Chemokine 71-Plex Discovery Assay Array (HD71; Eve Technologies, Calgary, Canada), according to the manufacturer’s instructions. Raw concentration data were imported into R, analyte columns were converted to numeric values, manufacturer-provided average rows were excluded, and technical duplicates were averaged to obtain one value per analyte per sample. Values missing in both technical replicates remained missing; no imputation was performed. The final Luminex dataset included 31 maternal plasma and 31 cord plasma samples from 34 participants, including 28 participants with paired maternal and cord plasma samples. Maternal and cord plasma were analyzed separately. Analyte concentrations were transformed as log_2_ (concentration +1), and associations with maternal third-trimester BMI were evaluated using analyte-specific linear models. Effect estimates and 95% confidence intervals were reported per 5-unit increase in BMI. Benjamini–Hochberg correction was performed separately across the 71 analytes within each plasma compartment. BMI tertiles were defined once at the participant level, and used only for visualization and exploratory pairwise contrasts. Because no analyte remained significant after multiple-testing correction, all nominal Luminex associations were considered hypothesis-generating.

### 2.10 Estimated cell-composition analyses

Relative PBMC and CBMC cell fractions were estimated using CIBERSORTx (relative mode, no quantile normalization) (Newman et al., 2019) with the LM22 (Newman et al., 2015) and a custom 12-class neonatal matrix (GSE212309) (Yin et al., 2024), respectively. CBMC outputs lacked S-mode batch correction due to non-convergence and were interpreted cautiously. Cell types detected in <50% of samples were excluded from statistical testing. Lacking a matched placental single-cell reference, placental composition was approximated using mean z-scores of variance-stabilized expression for five marker sets (trophoblast, stromal, endothelial, Hofbauer macrophage, and erythroid) (Pique-Regi et al., 2019). Associations between composition estimates (fractions or marker scores) and continuous third-trimester BMI (per 5-unit increase) were evaluated via linear regression, adjusting for sequencing batch when estimable, followed by Benjamini-Hochberg correction. For sensitivity analyses, composition PC1 was derived from centered log-ratio-transformed CIBERSORTx fractions or standardized placental marker scores to assess robustness to major compositional variation.

### 2.11 Clinical characteristics and focused sensitivity analyses

Descriptive clinical comparisons across participant-level third-trimester BMI tertiles (low [n = 7], middle [n = 7], and high [n = 6]) used Kruskal–Wallis tests for continuous variables (reported as median [Q1, Q3]) and Fisher’s exact tests for categorical variables (n [%]). All P-values were unadjusted and considered purely descriptive. Focused sensitivity analyses evaluated the robustness of key BMI-associated genes (9 PBMC, 14 placenta, 7 CBMC; Supplementary Table S8). Starting from the primary batch-adjusted DESeq2 model, we iteratively added maternal age, gestational age, infant sex, or a major complication composite (gestational diabetes, hypertension, and preeclampsia) individually and jointly. Further models adjusted for individual complications or compartment-specific composition PC1. Valid models (requiring a full-rank design matrix and ≥3 residual degrees of freedom) were assessed for consistency in BMI coefficient direction and magnitude. Given the limited sample size and potential for mediation, these served strictly as exploratory robustness checks rather than definitive control of confounding.

### 2.12 Placental RT-qPCR validation

Targeted quantitative reverse transcription PCR (RT-qPCR) was performed on available villous placental tissue from 15 donors spanning a broad range of third-trimester BMI values. Two additional samples were excluded from the final analysis due to substantial variance between technical replicates. Reactions were performed in technical duplicates using Bio-Rad SYBR Green Master Mix and custom primers (Integrated DNA Technologies) for *FDXR* (forward: TAC​AAC​GGG​CTT​CCT​GAG​AAC​C, reverse: GTC​CGT​TCT​CTG​GCA​CAA​AAG​G), *NMNAT2* (forward: GTA​GTG​ACC​TGC​TGG​AGT​CCT​T, reverse: ATG​ATT​CGG​TCT​GTG​TCG​GCT​G), and *ZFP57* (forward: CCA​TCT​CCC​AAA​CAC​AGA​AGG​C, reverse: CAA​GGA​AGA​CCT​TGT​CAT​CAC​TAG). *β-actin* (*ACTB*; forward: CAC​CAT​TGG​CAA​TGA​GCG​GTT​C, reverse: AGG​TCT​TTG​CGG​ATG​TCC​ACG​T) served as the endogenous reference gene. Relative expression was calculated as ΔCt = mean Ct_target − mean Ct_ACTB, with lower ΔCt values indicating higher relative target-gene abundance. Associations between continuous maternal third-trimester BMI and donor-level mean ΔCt values were evaluated using two-sided Pearson correlation analyses implemented in R.

### 2.13 Statistical analysis

All analyses were performed in R (R Core Team, 2024). Primary transcriptomic and Luminex models evaluated third-trimester BMI continuously. Benjamini–Hochberg FDR correction was applied separately within each compartment (Benjamini and Hochberg, 1995). Significance thresholds were FDR <0.10 for exploratory RNA-seq differential expression and FDR <0.05 for Hallmark enrichment. Exploratory Luminex analyses applied FDR correction across the 71 analytes per plasma compartment; because no analyte achieved FDR <0.05, nominal P < 0.05 was used solely for visualization. For descriptive comparisons and visualization—not primary association testing—BMI tertiles were defined separately within PBMC, placenta, and CBMC datasets due to varying sample availability, but defined once at the participant level for plasma analyses. Tertile ranges and sample sizes are detailed in the figure legends. Finally, associations between continuous BMI and placental RT-qPCR ΔCt values were evaluated using two-sided Pearson correlations.

## 3 Results

### 3.1 Study design and cohort characteristics

We conducted a cross-sectional multi-compartment study of 20 pregnant participants to evaluate associations between maternal third-trimester BMI and molecular profiles across the maternal–placental–fetal axis. Maternal peripheral blood, term placental villous tissue, umbilical cord blood, maternal plasma, and cord plasma were collected at delivery when available. The primary transcriptomic analyses modeled third-trimester BMI as a continuous variable, with BMI tertiles used only for visualization and interpretation of expression and module patterns.

Clinical and demographic characteristics of the transcriptomic cohort are summarized in Table 1. Participants self-identified as Black or African American; 19 were non-Hispanic, and one was Hispanic. Third-trimester BMI was enriched toward higher values, with a median BMI of 34.4 kg/m^2^ (Q1, 30.3; Q3, 40.1). All deliveries occurred at term by uncomplicated cesarean section, with a median gestational age of 39 weeks. The sex of the infants was 10 female and 10 male newborns. RNA-seq data were available for 17 maternal PBMC samples, 18 CBMC samples, and 14 placental samples. Exploratory Luminex profiling was performed in a larger available plasma cohort of 34 pregnancies, comprising 31 maternal plasma and 31 cord plasma samples, including 28 paired maternal–cord plasma samples (Supplementary Table S1).

**TABLE 1:** Clinical and demographic characteristics of the transcriptomic cohort.

Characteristic	Transcriptomic cohort (N = 20)^1^
Race	​
Black or african american	20 (100.0%)
Ethnicity	​
Hispanic	1 (5.0%)
Non-hispanic	19 (95.0%)
Maternal third-trimester BMI, kg/m^2^	34.4 (30.3, 40.1)
Systolic blood pressure, mmHg	124.0 (108.5, 131.0)
Diastolic blood pressure, mmHg	76.5 (71.0, 81.5)
Blood glucose, mg/dL	85.0 (74.5, 99.5)
Missing	4
Gestational age at delivery, weeks	39.0 (39.0, 39.0)
Term delivery	​
Term	20 (100.0%)
Infant sex	​
Female	10 (50.0%)
Male	10 (50.0%)
Birth weight, g	3,160 (2,963, 3,390)
Birth length, inches	19.5 (19.0, 20.0)
PBMC RNA-seq available	17 (85.0%)
CBMC RNA-seq available	18 (90.0%)
Placental RNA-seq available	14 (70.0%)

^1^n (%); median (Q1, Q3).

Values are shown as median (Q1, Q3) for continuous variables and n (%) for categorical variables.

Clinical and neonatal characteristics were compared across participant-level third-trimester BMI tertiles (low [n = 7], middle [n = 7], and high [n = 6]; Supplementary Table S7). Although no descriptive comparison yielded a nominal P-value <0.05, this does not establish clinical equivalence due to the small cohort, missing observations, and sparse pregnancy complications. Specifically, the cohort included only one case of gestational diabetes, three of hypertension, and zero of clinically recorded preeclampsia.

### 3.2 Global transcriptomic variation is dominated by distinct compartment identity across the maternal–placental–fetal axis

To define the global structure of the multi-compartment transcriptomic dataset, we first performed principal component analysis (PCA) across all available maternal PBMC, placental, and CBMC RNA-seq samples. Samples were separated primarily by compartment (Supplementary Figure S1B), indicating that compartmental identity was the dominant source of transcriptomic variation. Placental samples formed a distinct cluster, whereas PBMC and CBMC samples were more closely related to each other but remained separable, consistent with their shared immune-cell lineage and distinct maternal versus fetal sources.

Because compartment identity accounted for the largest transcriptomic differences across the dataset, subsequent BMI-associated analyses were performed separately within each compartment. These compartment-specific analyses identified BMI-associated gene expression programs in maternal PBMCs, placental tissue, and CBMCs.

### 3.3 Higher third-trimester BMI was associated with compartment-specific transcriptomic remodeling

We next evaluated whether increasing third-trimester BMI was associated with compartment-specific transcriptional changes across maternal PBMCs, placenta, and CBMCs. Differential expression analysis was performed separately in each compartment, with third-trimester BMI treated as a continuous variable. Significant BMI-associated genes were identified in all three compartments, with the largest number detected in placenta tissue, followed by PBMCs, and CBMCs (Table 2). The direction and magnitude of BMI-associated gene changes differed across compartments, consistent with compartment-specific transcriptomic patterns rather than a uniform response across the maternal–placental–fetal axis.

**TABLE 2:** Differentially expressed Genes Associated with Maternal third-trimester BMI by compartment. Genes are shown from compartment-specific continuous BMI models. Direction indicates whether an association is positive or negative with increasing third-trimester BMI. log2FC values are shown per 1 standard deviation increase in BMI and rescaled per 5-unit increase in BMI. FDR values were calculated using the Benjamini–Hochberg method.

Tissue	Gene	Direction	log2FC per 1 SD BMI	log2FC per +5 BMI	P Value	FDR
CBMC	XG	Down	−2.911	−1.912	0.000000944	0.0142
	GPR21	Up	1.705	1.120	0.000002180	0.0164
	RBPMS2	Down	−1.569	−1.031	0.000005220	0.0261
	TACSTD2	Up	1.925	1.265	0.000008450	0.0317
	MFAP5	Up	2.438	1.601	0.000012900	0.0389
	SEC14L5	Down	−1.449	−0.952	0.000025900	0.0647
	DHRS9	Down	−0.879	−0.578	0.000039800	0.0853
PBMC	NIBAN3	Up	0.635	0.435	0.000002490	0.0266
	FCRL2	Up	0.736	0.504	0.000003680	0.0266
	PEG10	Up	0.937	0.642	0.000005270	0.0266
	IL1R2	Down	−2.984	−2.043	0.000008550	0.0324
	TARM1	Down	−2.791	−1.911	0.000012500	0.0359
	CMBL	Down	−1.165	−0.798	0.000014200	0.0359
	CH25H	Down	−2.832	−1.939	0.000024900	0.0540
	FCER2	Up	0.642	0.440	0.000039600	0.0751
	SCN3A	Up	1.055	0.722	0.000052700	0.0889
Placenta	ZFP57	Up	1.679	1.151	0.000001750	0.0213
	MGA	Down	−0.156	−0.107	0.000002980	0.0213
	PIKFYVE	Down	−0.141	−0.096	0.000011500	0.0486
	SOHLH2	Up	0.741	0.508	0.000017100	0.0486
	MACF1	Down	−0.254	−0.174	0.000017700	0.0486
	SETX	Down	−0.148	−0.101	0.000020400	0.0486
	MYCBP2	Down	−0.182	−0.125	0.000028800	0.0506
	CA2	Up	0.550	0.377	0.000029400	0.0506
	RAPGEF6	Down	−0.141	−0.097	0.000038100	0.0506
	SMG1	Down	−0.207	−0.142	0.000041100	0.0506
	NMNAT2	Up	0.889	0.609	0.000041400	0.0506
	AQP3	Up	1.270	0.870	0.000044500	0.0506
	VPS13B	Down	−0.239	−0.164	0.000046000	0.0506
	FDXR	Up	0.268	0.184	0.000084200	0.0820
	HECTD4	Down	−0.201	−0.138	0.000086100	0.0820
	MIB1	Down	−0.170	−0.116	0.000092000	0.0822
	CEP68	Down	−0.234	−0.160	0.000117,000	0.0987

#### 3.3.1 Maternal PBMCs showed a modest BMI-associated transcriptomic signature

In maternal PBMCs, continuous differential expression analysis identified a modest but distinct BMI-associated transcriptional signature (Figure 1A). The top BMI-associated genes showed coordinated expression gradients across samples ordered by third-trimester BMI, indicating structured transcriptional variation across the BMI range. Higher third-trimester BMI was associated with increased expression of PEG10, FCRL2, NIBAN3, FCER2, and SCN3A, whereas CMBL, IL1R2, TARM1, and CH25H were decreased. Representative effect estimates per 5-unit increase in third-trimester BMI included increased expression of PEG10 (log_2_FC = 0.64; FDR = 0.027) and decreased expression of IL1R2 (log_2_FC = −2.04; FDR = 0.032) (Table 2; Supplementary Table S2). These findings suggest that higher third-trimester BMI is associated with coordinated differences in gene expression in maternal circulating immune cells.

**FIGURE 1:**
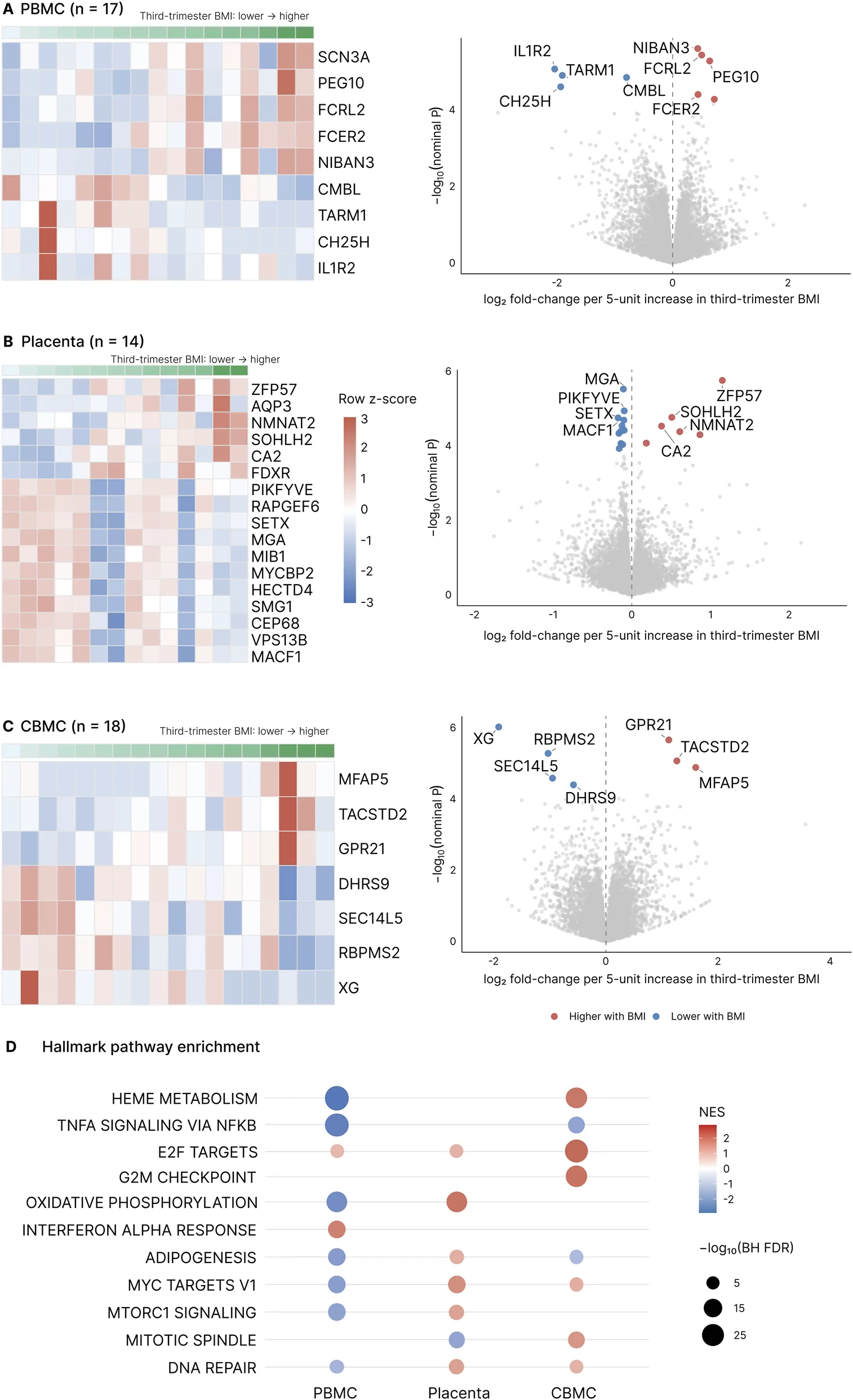
Third-trimester BMI-associated transcriptomic patterns across maternal PBMCs, placental tissue, and CBMCs. **(A–C)** Heatmaps show row-wise z-scored variance-stabilized expression for all genes meeting the compartment-specific Benjamini–Hochberg (BH) FDR threshold of 0.10 in PBMCs (9 genes; n = 17), placental tissue (17 genes; n = 14), and CBMCs (7 genes; n = 18). Genes were ordered from positive to negative estimated effects, and samples were ordered by continuous third-trimester BMI within each compartment. Heatmap values were capped at z scores of ±3 for visualization. Volcano plots show unshrunken DESeq2 estimates rescaled to represent the log_2_ fold-change per 5-unit increase in third-trimester BMI. Colored points indicate genes meeting BH FDR <0.10; up to four genes with the lowest FDR in each direction were labeled. **(D)** Hallmark dot plot showing the union of the five lowest-FDR pathways within each compartment among pathways meeting BH FDR <0.05. Color represents the normalized enrichment score (NES), and point size represents −log_10_(BH FDR), capped at 25 for visualization. Complete gene-level and Hallmark pathway results are provided in Supplementary Tables S2, S3, respectively.

#### 3.3.2 Placental tissue showed the most extensive BMI-associated transcriptomic signature

Placental transcriptomes exhibited the most extensive BMI-associated transcriptional signatures among the three compartments (Figure 1B). Genes positively associated with third-trimester BMI included ZFP57, SOHLH2, NMNAT2, AQP3, CA2, and FDXR, whereas MGA, PIKFYVE, MACF1, SETX, MYCBP2, VPS13B, RAPGEF6, and SMG1 were negatively associated. Representative effect estimates per 5-unit increase in third-trimester BMI included increased expression of ZFP57 (log_2_FC = 1.15; FDR = 0.021) and decreased expression of MGA (log_2_FC = −0.11; FDR = 0.021) (Table 2; Supplementary Table S2). These gene-level changes highlight candidate placental transcripts related to mitochondrial/redox regulation, transcriptional control, substrate transport, and cytoskeletal or developmental signaling.

#### 3.3.3 CBMCs showed a distinct BMI-associated transcriptomic signature

CBMC transcriptomes also showed a coherent, although smaller, BMI-associated transcriptional signature (Figure 1C). Higher third-trimester BMI was associated with increased expression of GPR21, TACSTD2, and MFAP5, whereas XG, RBPMS2, SEC14L5, and DHRS9 were decreased. Representative effect estimates per 5-unit increase in third-trimester BMI included increased expression of GPR21 (log_2_FC = 1.12; FDR = 0.016) and decreased expression of XG (log_2_FC = −1.91; FDR = 0.014) (Table 2; Supplementary Table S2). The top BMI-associated CBMC genes exhibited coordinated expression patterns across samples, with positively associated genes tending to be more highly expressed at the upper end of the third-trimester BMI range and negatively associated genes showing the opposite pattern, despite some residual inter-sample heterogeneity. These exploratory results indicate that third-trimester BMI is associated with candidate remodeling signatures in the fetal immune compartment at birth.

#### 3.3.4 Focused clinical sensitivity analyses of key BMI-associated genes

Focused sensitivity analyses evaluated whether key gene-level BMI associations were robust to clinical adjustment (Supplementary Table S8; Supplementary Figure S3). All estimable one-covariate models retained the direction of the primary BMI coefficient across PBMCs (9 of 9), placenta (14 of 14), and CBMCs (7 of 7). Exploratory joint models were estimable in placenta and CBMCs, retaining all directional associations; the corresponding PBMC joint model was non-estimable due to lack of gestational-age variation. Models adjusted for gestational diabetes or preeclampsia could not be evaluated due to sparse or absent cases (1 case and 0 cases, respectively). Models incorporating gestational age exhibited poor numerical conditioning. While effect sizes varied and significance was not uniformly retained, these results support directional consistency but do not demonstrate definitive control of confounding.

#### 3.3.5 Estimated cell composition and associations with third-trimester BMI

CIBERSORTx cell-fraction estimates (PBMC n=17 , CBMC n=18 ) and placental marker scores ( n=14 ) showed no associations with third-trimester BMI after Benjamini–Hochberg correction (Supplementary Table S9; Supplementary Figure S2). In PBMCs, a nominal positive association was observed for naive B cells ( β=0.0094 ; FDR = 0.273), whereas no CBMC fractions were nominally associated. Because the custom CBMC reference showed a lower technical fit than the PBMC LM22 matrix (median mixture-fit correlations: 0.530 vs. 0.890), the resulting fetal cell-fraction estimates were interpreted with caution. In placental tissue, marker scores showed no significant associations. Crucially, adjustment for compartment-specific composition PC1 preserved the direction of all key-gene associations (Supplementary Table S8; Supplementary Figure S3). While major estimated compositional shifts did not fully account for these findings, bulk RNA-seq data cannot definitively isolate cell-intrinsic transcriptional regulation from cellular proportions or activation states.

### 3.4 Integrated hallmark pathway analysis reveals divergent BMI-associated pathway patterns across compartments

To determine whether BMI-associated transcriptional changes converged on shared or compartment-specific biological programs, we integrated Hallmark GSEA results across PBMCs, placental tissue, and CBMCs. This analysis revealed divergent pathway directionality across compartments rather than a uniform BMI-associated response (Figure 1D).

Several pathways showed opposite patterns between placental and immune-cell compartments. Oxidative phosphorylation was enriched in placental tissue but depleted in PBMCs, suggesting increased placental mitochondrial activity alongside reduced metabolic pathway activity in maternal circulating immune cells. Similarly, adipogenesis was positively associated with BMI in placental tissue but negatively associated in PBMCs and CBMCs. Interferon gamma response also showed compartment-specific directionality, with positive enrichment in PBMCs but negative enrichment in placental tissue and CBMCs.

Together, these results indicate that increasing third-trimester BMI is associated with coordinated but compartment-specific pathway remodeling across the maternal–placental–fetal axis. Maternal PBMCs showed selective immune activation with broader metabolic attenuation, placental tissue showed metabolic and stress-response activation, and CBMCs showed proliferative remodeling with relative attenuation of inflammatory and immune-response pathways.

### 3.5 Exploratory co-expression network analysis nominates BMI-associated inflammatory, vascular, and extracellular matrix modules

To evaluate whether BMI-associated transcriptional signals were reflected in coordinated gene-expression programs, we performed compartment-specific exploratory co-expression network analysis using CEMiTool (Figures 2A–C). Modules were identified independently within maternal PBMCs, placental tissue, and CBMCs, and module eigengenes were tested for association with continuous third-trimester BMI. Module activity was also summarized across third-trimester BMI tertiles for visualization only. Reactome over-representation analysis and STRING-supported PPI subnetworks were used to annotate selected modules and identify candidate hub genes (Figure 2; Supplementary Figure S4). Because of the modest sample size, these analyses were interpreted as hypothesis-generating and used to provide network-level context for the differential expression and Hallmark pathway findings.

**FIGURE 2:**
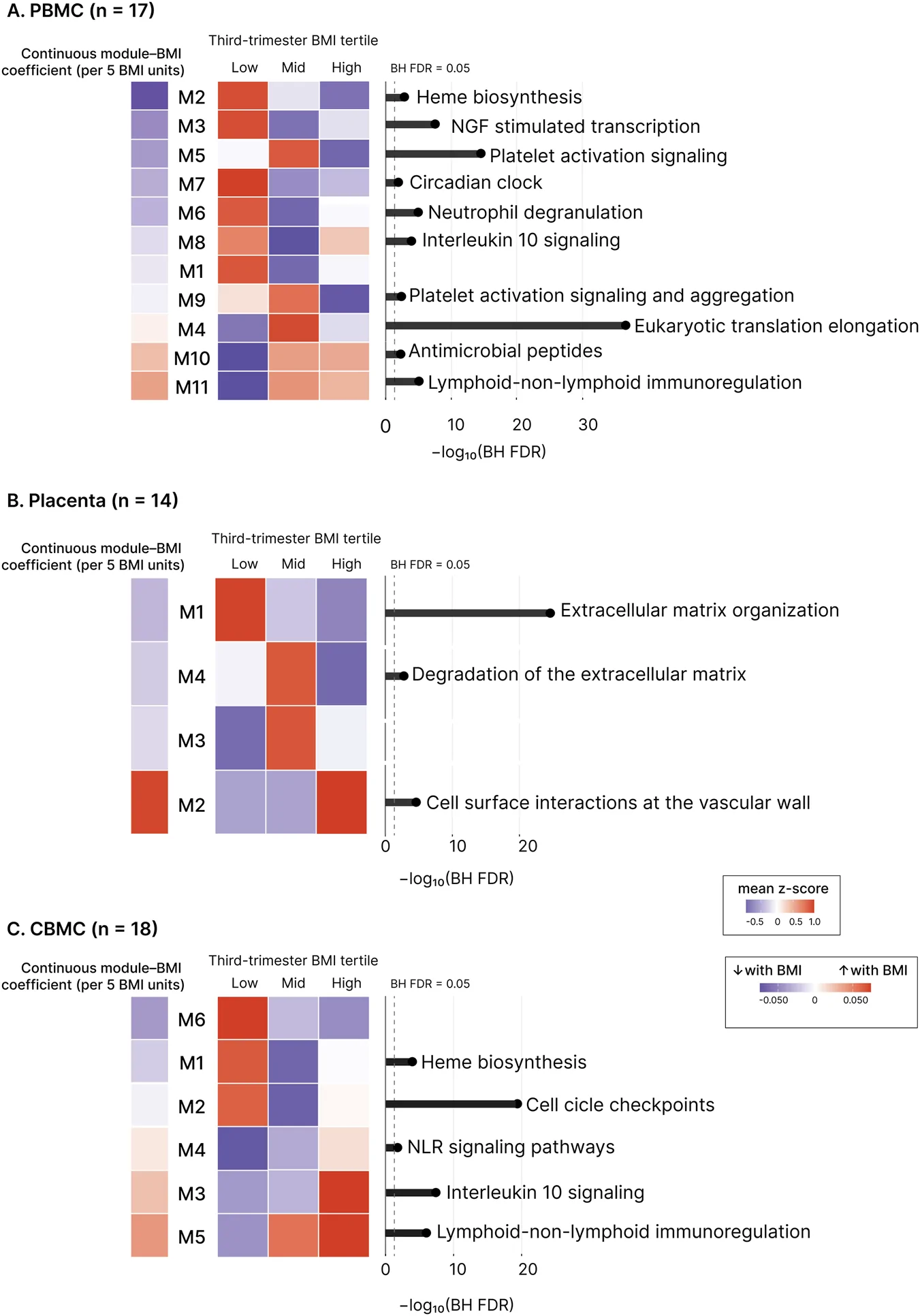
Exploratory co-expression modules and their associations with third-trimester BMI. CEMiTool co-expression analyses are shown for **(A)** PBMCs (n = 17) **(B)** placental tissue (n = 14), and **(C)** CBMCs (n = 18). The left color strip represents the continuous module–BMI regression coefficient per 5-unit increase in third-trimester BMI, with purple and orange indicating negative and positive coefficients, respectively. The central heatmaps show mean standardized module eigengene scores across low, middle, and high third-trimester BMI tertiles. Tertiles were defined separately within each compartment because sample availability differed and were used only for visualization; compartment-specific ranges and sample sizes are reported in Supplementary Table S4. The right panels show a representative Reactome pathway for each module when available. Line length and point position represent −log_10_(BH FDR), and the dashed line indicates BH FDR = 0.05. Detailed module membership, eigengene associations, enrichment results, and network annotations are provided in Supplementary Tables S4, S5; Supplementary Figure S4. These network results are exploratory.

#### 3.5.1 PBMC modules highlight inflammatory-regulatory and platelet/integrin-associated programs

In maternal PBMCs, selected BMI-related modules suggested inflammatory regulation and vascular–immune interface biology (Figure 2A). Module M8 showed a non-linear pattern across third-trimester BMI tertiles, with relatively higher expression in the low BMI tertile, reduced expression in the mid BMI tertile, and partial recovery in the high third-trimester BMI tertile. Functional annotation linked this module to interleukin-10 signaling, and the corresponding PPI subnetwork included high-degree inflammatory and chemokine-related hubs such as IL1B, CXCL8, CXCR1, NFKBIA, PTGS2, CCL3L1, FCGR3B, and MMP9. Module M5 was enriched for platelet activation, signaling, aggregation, and adhesion-related pathways, with a PPI subnetwork centered on platelet and integrin-associated hubs including ITGA2B, SELP, GP9, GP1BA, GP6, ITGB3, PF4, and CLEC1B. These exploratory module patterns are consistent with third-trimester BMI-related PBMC remodeling involving coordinated inflammatory-regulatory and platelet/integrin-associated programs.

#### 3.5.2 Placental modules implicate extracellular matrix, integrin, and vascular-interface remodeling

In placental tissue, exploratory co-expression analysis highlighted BMI-associated modules related to extracellular matrix, integrin, vascular-interface, endocrine, and erythroid/oxygen-transport biology (Figure 2B). Module M1 showed higher module activity in the low third-trimester BMI tertile and lower activity in the mid and high BMI tertiles, suggesting reduced ECM/integrin-associated network activity across higher third-trimester BMI strata. This module was enriched for extracellular matrix organization and integrin cell-surface interactions, with a STRING-supported PPI subnetwork containing high-degree vascular, immune-interface, and extracellular matrix-associated nodes, including CD4, ITGAM, KIT, FGF2, MMP9, KDR, PTPRC, and COL1A1. In contrast, Module M2 showed higher activity in the high BMI tertile and was enriched for cell-surface interactions at the vascular wall and erythroid/oxygen-transport-related pathways. Its PPI subnetwork included placental endocrine/interface-associated and erythroid-related nodes such as TGFB1, LEP, PSG1, PSG3, CSH1, CSH2, ERBB2, and AHSP. These exploratory network results provide candidate evidence suggesting that increasing third-trimester BMI is associated with coordinated variation in placental extracellular matrix, integrin, vascular-interface, endocrine, and oxygen-transport-related programs.

#### 3.5.3 CBMC modules suggest candidate immune, cytotoxic, and heme-related programs

In CBMCs, co-expression modules highlighted immune and cytotoxic programs associated with higher third-trimester BMI, alongside relative attenuation of heme-related modules (Figure 2C). Module M3 showed higher expression in the high BMI tertile and was enriched for interleukin-10 signaling, with a PPI subnetwork organized around inflammatory and stress-response hubs including IL1B, CXCL8, ICAM1, PTGS2, HIF1A, FOSB, EGR1, and EGR2. Module M5 was also increased at higher third-trimester BMI and captured a cytotoxic/lymphoid-associated program, with prominent PPI and co-expression hubs including GZMB, KLRD1, PRF1, GZMA, CCL5, FCGR3B, GNLY, GZMH, NKG7, IL2RB, and TBX21. In contrast, heme biosynthesis-associated modules were relatively higher in the low-BMI tertile and lower in the mid- and high-BMI tertiles. These findings support BMI-associated remodeling of fetal immune-cell programs at the network level.

Together, the co-expression analysis provided an exploratory network-level framework that was broadly consistent with the gene-level and Hallmark pathway results. Rather than serving as a confirmatory analysis, these modules highlighted candidate coordinated programs that may contribute to BMI-related remodeling across the maternal–placental–fetal axis, including inflammatory-regulatory and platelet/integrin-associated programs in maternal PBMCs, extracellular matrix, vascular-interface, and placental endocrine-associated programs in placental tissue, and inflammatory, cytotoxic/lymphoid, and heme-related programs in CBMCs.

### 3.6 Plasma cytokine profiling supports compartment-specific BMI-associated immune remodeling

Exploratory Luminex profiling was performed in 31 maternal plasma and 31 cord plasma samples from 34 participants, including 28 participants with paired maternal and cord plasma samples (Figure 3). In analyte-specific continuous models, four maternal-plasma analytes and three cord-plasma analytes showed nominal associations with maternal third-trimester BMI. However, none remained significant after Benjamini–Hochberg correction across the 71 analytes tested separately within each plasma compartment (minimum FDR: maternal plasma, 0.577; cord plasma, 0.547; Supplementary Table S6).

**FIGURE 3:**
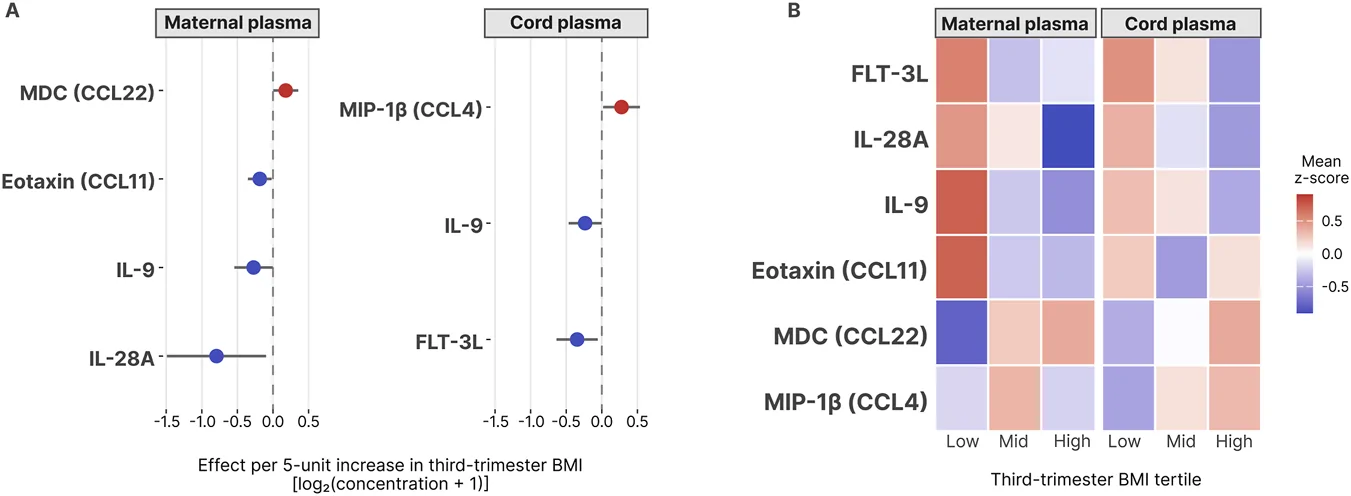
Exploratory associations between maternal third-trimester BMI and plasma cytokines and chemokines. Luminex profiling included 31 maternal plasma and 31 cord plasma samples from 34 participants. **(A)** Effect estimates from analyte-specific continuous BMI models performed separately in maternal and cord plasma. Points represent the estimated change in log_2_ (concentration +1) per 5-unit increase in BMI, and horizontal lines represent 95% confidence intervals. Only analytes with nominal P < 0.05 are displayed. **(B)** Mean standardized abundance of the corresponding analytes across participant-level BMI tertiles. Tertiles were defined once among the 34 participants with at least one available Luminex sample: low, 24.0–31.2 kg/m^2^ (n = 12); mid, 31.3–36.6 kg/m^2^ (n = 11); and high, 37.2–49.7 kg/m^2^ (n = 11). Available sample numbers were maternal plasma: low n = 9, middle n = 11, and high n = 11; and cord plasma: low n = 11, mid n = 9, and high n = 11. Benjamini–Hochberg correction was applied separately across 71 analytes in each compartment. None of the displayed associations remained significant after multiple-testing correction; the results are therefore exploratory and hypothesis-generating.

In maternal plasma, each 5-unit increase in third-trimester BMI was nominally associated with higher MDC/CCL22 (β = 0.18; 95% CI, 0.004–0.35) and lower IL-28 A (β = −0.79 log_2_ units; 95% CI, −1.49 to −0.10), Eotaxin/CCL11 (β = −0.19; 95% CI, −0.35 to −0.02), and IL-9 (β = −0.27; 95% CI, −0.54 to −0.003). In cord plasma, each 5-unit increase in third-trimester BMI was nominally associated with higher MIP-1β/CCL4 (β = 0.28; 95% CI, 0.02–0.54) and lower FLT-3L (β = −0.35; 95% CI, −0.64 to −0.06) and IL-9 β = −0.23; 95% CI, −0.47 to −0.003).

Descriptive visualization across participant-level third-trimester BMI tertiles suggested that analyte patterns were not uniformly monotonic (Figure 3B). Complete results from the primary continuous-BMI models and secondary descriptive tertile analyses are provided in Supplementary Table S6. Given the absence of FDR-significant associations and the modest compartment-specific sample sizes, these findings should be interpreted as exploratory and hypothesis-generating rather than as evidence of definitive BMI-associated differences in plasma analyte concentrations.

### 3.7 Exploratory qRT-PCR evaluation of selected placental transcripts

To provide supportive evaluation of selected placental RNA-seq findings, qRT-PCR was performed on villous placental tissue from 15 donors spanning a broad range of third-trimester BMI values. Expression of FDXR, NMNAT2, and ZFP57 was quantified relative to β-actin and expressed as donor-level mean ΔCt values calculated from raw technical replicate Ct measurements. Third-trimester BMI was inversely associated with ΔCt values for FDXR (Pearson r = −0.57, p = 0.028), NMNAT2 (r = −0.71, p = 0.003) and ZFP57 (r = −0.68, p = 0.005) (Supplementary Figure S5). Because lower ΔCt values reflect higher target transcript abundance relative to β-actin, these exploratory findings were consistent with higher normalized placental expression of these transcripts with increasing third-trimester BMI.

## 4 Discussion

Maternal metabolic status is increasingly recognized as an important regulator of placental function and fetal developmental biology. However, the molecular pathways associated with third-trimester BMI across maternal, placental, and fetal compartments remain incompletely defined (Catalano and Shankar, 2017; Leddy et al., 2008; Monaco-Brown and Lawrence, 2022; Gaillard et al., 2013). In this exploratory study, we integrated transcriptomic profiling of maternal PBMCs, placental tissue, and CBMCs, complemented by multiplex cytokine profiling of maternal and cord plasma, to characterize molecular signatures associated with third-trimester BMI across the maternal–placental–fetal axis. We found that increasing third-trimester BMI was not associated with a uniform inflammatory signature across compartments. Instead, higher third-trimester BMI was linked to distinct but coordinated molecular programs involving maternal immune-metabolic remodeling, placental metabolic and oxidative-stress adaptation, and fetal immune-developmental changes at birth.

This integrated pattern suggests that BMI-associated molecular changes are distributed across the maternal–placental–fetal axis rather than confined to a single tissue. The placenta showed the largest BMI-associated transcriptional signature, consistent with its central role as a metabolic, endocrine, vascular, and immunological interface between maternal physiology and fetal development (Howell and Powell, 2017; Burton and Fowden, 2015; Monaco-Brown and Lawrence, 2022; Kelly et al., 2020; Jansson and Powell, 2013; Burton et al., 2016; Brombach et al., 2022). At the same time, maternal PBMC and CBMC signatures indicated that both maternal and fetal immune compartments were remodeled in association with third-trimester BMI, supporting a model in which maternal metabolic status is reflected across interconnected immune and placental pathways.

In maternal PBMCs, higher third-trimester BMI was associated with selective enrichment of interferon-related pathways together with broader attenuation of metabolic, inflammatory, and redox-related programs. This pattern suggests that circulating maternal immune cells do not simply adopt a globally activated inflammatory state in association with higher third-trimester BMI. Rather, they appear to undergo selective immune activation within a broader context of immune-metabolic attenuation. This interpretation is consistent with emerging evidence that maternal obesity can alter immune-cell plasticity, bioenergetic function, and inflammatory responsiveness during pregnancy (Fantuzzi, 2005; Xu et al., 2003; Sureshchandra et al., 2021; Sureshchandra et al., 2018; Gurung et al., 2024). In particular, Sureshchandra and colleagues showed that pregravid obesity disrupts the normal functional, genomic, epigenetic, and metabolic trajectories of maternal blood monocytes during pregnancy, resulting in a phenotype more consistent with maladaptive immune reprogramming than with simple hyperactivation (Sureshchandra et al., 2021).

At the gene level, increased expression of FCER2 and FCRL2 was compatible with selective adaptive immune features, particularly B-cell-associated biology, whereas reduced IL1R2 and CH25H suggested attenuation of selected innate immune regulatory and host-defense pathways (Zhao et al., 2020; Peters et al., 2013). However, these individual genes are best interpreted within the broader pathway and network context. Exploratory co-expression modules enriched for IL-10 signaling and platelet/integrin-associated pathways provided supportive evidence that BMI-related PBMC remodeling may involve coordinated inflammatory-regulatory and vascular–immune interface programs rather than isolated gene-level changes, with PPI subnetworks highlighting inflammatory and platelet/integrin-associated hubs. These findings support a model in which higher third-trimester BMI was associated with an altered maternal immune-cell state characterized by selective immune signaling superimposed on broader immune-metabolic attenuation.

Placental transcriptomes exhibited the strongest BMI-associated molecular remodeling in this cohort. The placental signature was dominated by metabolic and stress-response pathways, including oxidative phosphorylation, reactive oxygen species signaling, unfolded protein response, MTORC1 signaling, adipogenesis, and DNA repair. These findings are consistent with increased placental metabolic demand and cellular stress adaptation in pregnancies with higher third-trimester BMI. A directly relevant comparison is the study by Sureshchandra et al. (Sureshchandra et al., 2018), which used bulk RNA sequencing of term placental tissue to compare pregnancies with pregravid obesity and lean controls and reported altered expression of genes involved in nutrient transport and immune function. Our findings show partial biological overlap with that study but additionally highlight mitochondrial/redox, extracellular-matrix, vascular-interface, and endocrine-associated programs, without evidence of uniform inflammatory activation. More broadly, previous studies have associated maternal obesity with altered placental morphology, nutrient transport, inflammatory signaling, mitochondrial function, and cellular stress responses (Howell and Powell, 2017; Monaco-Brown and Lawrence, 2022; Kelly et al., 2020; Aye et al., 2014; Myatt and Maloyan, 2016; Santos-Rosendo et al., 2020; Dimasuay et al., 2016; Burton et al., 2016; Brombach et al., 2022; Mele et al., 2014; Hebert and Myatt, 2021; Zhang et al., 2024; Mandò et al., 2018). Nevertheless, the present findings should be compared qualitatively rather than interpreted as a direct replication because Sureshchandra et al. evaluated categorical pregravid obesity, whereas we modeled third-trimester BMI continuously, and the studies differed in cohort composition, placental sampling, and analytical approaches. Because these data were generated from bulk placental tissue, these signatures may reflect transcriptional changes within trophoblast, stromal, endothelial, or immune cells, differences in cellular composition or both.

Several BMI-associated placental genes supported this interpretation. Increased expression of FDXR and NMNAT2 is consistent with activation of mitochondrial and redox-regulatory pathways, while increased AQP3 and CA2 suggests altered substrate transport and acid–base regulatory processes at the maternal–fetal interface (Imamichi et al., 2014; Liu and Chen, 2002; Mayer et al., 2010; Xie et al., 2020; Esbaugh and Tufts, 2006; Reijnders et al., 2018; Zhang et al., 2020; Seo et al., 2018). Increased ZFP57, a regulator of genomic imprinting, may indicate altered transcriptional or epigenetic regulatory activity in the setting of maternal metabolic stress (Quenneville et al., 2011; Messerschmidt et al., 2012; Takahashi et al., 2019; Su et al., 2016; Argentato et al., 2023; Hjort et al., 2022). The exploratory qRT-PCR findings provided supportive evidence for increased relative placental abundance of FDXR, NMNAT2, and ZFP57 with increasing third-trimester BMI. Together, these results support a model in which higher maternal BMI is associated with coordinated placental metabolic, redox, endocrine, immune-regulatory, and epigenetic changes that may influence trophoblast function, nutrient sensing and transport, vascular adaptation, and signaling at the maternal-fetal interface. However, these findings remain exploratory and do not establish the cellular source, functional consequences, or clinical significance of the observed transcript changes. Larger independent cohorts and cell-specific functional studies will be needed to determine whether these BMI-associated placental pathways contribute to altered placental function or fetal developmental programming.

In parallel with these metabolic and stress-response signatures, exploratory placental co-expression analysis highlighted candidate modules related to extracellular matrix organization, integrin interactions, vascular/interface pathways, and placental endocrine-associated remodeling. Downregulation of genes involved in cytoskeletal organization and developmental signaling, including MACF1 and RAPGEF6, suggests altered regulation of cell adhesion, cytoskeletal dynamics, and tissue organization (Hjort et al., 2022; Chen et al., 2006; Knöfler et al., 2019; Maeta et al., 2016). These processes are relevant to placental morphogenesis, trophoblast behavior, and vascular remodeling at the maternal–fetal interface (Beneventi et al., 2023; Burton et al., 2016; Knöfler et al., 2019). The placental results support a model in which higher third-trimester BMI is associated with a metabolically active but structurally remodeled placenta, characterized by stress-response activation alongside alterations in extracellular matrix, integrin, vascular-interface, and placental endocrine-associated biology.

In the fetal compartment, CBMC transcriptomes showed a coherent BMI-associated signature that differed from both maternal PBMCs and placental tissue. The dominant CBMC pattern was not broad fetal inflammatory activation, but rather proliferative and immune-developmental remodeling with relative attenuation of inflammatory signaling. This pattern included enrichment of cell-cycle and proliferative programs together with reduced activity of several inflammatory and immune-response pathways. These findings are consistent with prior evidence that maternal obesity can influence fetal gene expression and immune response programs detectable in umbilical cord blood (Edlow et al., 2016; Sureshchandra et al., 2023). Edlow and colleagues used cord blood transcriptomic profiling to identify fetal gene-expression differences associated with maternal obesity at term, supporting the concept that cord blood can capture molecular effects of the intrauterine metabolic environment (Edlow et al., 2016). In addition, Sureshchandra and colleagues showed that maternal obesity dysregulates fetal monocyte responses to bacterial and viral stimulation, further supporting the idea that fetal immune cells can be functionally remodeled in association with maternal metabolic status (Sureshchandra et al., 2023).

At the gene level, increased expression of GPR21, TACSTD2, and MFAP5 may reflect immune-metabolic sensitivity, developmental signaling, and extracellular matrix remodeling in the fetal compartment (Osborn et al., 2012; Gardner et al., 2012; Kroeze et al., 2003; Lumeng and Saltiel, 2011; Leonard et al., 2016; Groom et al., 2012; Turcot et al., 2012; Vaittinen et al., 2015; Adapala et al., 2012). Conversely, reduced RBPMS2 levels may suggest altered RNA regulatory programs relevant to cardiovascular and developmental biology (Gan et al., 2022; Akerberg et al., 2019; Akerberg et al., 2022). As with the maternal and placental compartments, however, the overriding interpretation emerges from the convergence of gene- and pathway-level findings and co-expression analyses. CBMC co-expression modules provided candidate network-level support for interleukin signaling, cytotoxic/lymphoid-associated programs, and heme-related biology, with PPI subnetworks highlighting inflammatory, stress-response, and cytotoxic/lymphoid hubs.

The exploratory cell-composition analyses provided additional context for interpreting the bulk transcriptomic findings. No estimated PBMC or CBMC cell fraction or placental marker score remained associated with third-trimester BMI after multiple-testing correction. The nominal increase in the estimated PBMC naive B-cell fraction may be compatible with the B-cell-associated transcriptomic signals involving FCER2 and FCRL2; however, this association did not survive FDR correction and should not be interpreted as definitive evidence of compositional change. Moreover, adjustment for the dominant estimated composition axis preserved the direction of all estimable key-gene associations. These findings suggest that major estimated compositional differences did not fully account for the selected transcriptomic associations under the approaches used. Nevertheless, they do not establish that the observed differences represent transcriptional regulation within individual cell types.

Exploratory plasma cytokine profiling suggested a small number of nominal, compartment-specific associations with maternal third-trimester BMI, but no analyte remained significant after correction for testing 71 analytes within each compartment. The corresponding effect estimates were modest and had relatively wide confidence intervals. Maternal plasma showed a nominal positive association with MDC/CCL22 and negative associations with IL-28A, Eotaxin/CCL11, and IL-9, whereas cord plasma showed a nominal positive association with MIP-1β/CCL4 and negative associations with FLT-3L and IL-9. The differing directions and tertile patterns did not support a uniform systemic inflammatory response to increasing BMI. These results should therefore be regarded as exploratory signals requiring replication in larger independent cohorts.

A major strength of this study is the multi-compartment profiling of maternal blood, placental tissue, cord blood, and available plasma immune mediators collected within the same study framework, enabling a systems-level view of BMI-associated remodeling across the maternal–placental–fetal axis. By integrating differential expression, Hallmark pathway enrichment, co-expression network analysis, and cytokine profiling, this study captures transcriptional programs and provides an exploratory assessment of circulating immune mediators associated with third-trimester BMI across maternal, placental, and fetal compartments. Although this design provides a foundation for future paired dyad-level analyses, the modest cohort size and incomplete sample availability across compartments limited formal testing of maternal–placental–fetal mediation or coupling in the present study. Another strength is the analysis of a cohort composed of Black or African American participants, a population that remains underrepresented in many molecular studies of pregnancy biology.

Several limitations should be considered. First, the modest sample size limits statistical power, meaning all transcriptomic, network, and cytokine findings must be interpreted as exploratory rather than definitive. Furthermore, sparse pregnancy complications and limited variation in clinical factors restricted reliable covariate adjustment; while sensitivity models suggested directional robustness, definitive multivariable control for clinical confounding was not possible. Second, we evaluated third-trimester BMI, which reflects pre-pregnancy adiposity, gestational weight gain, and fetal/placental mass. Without complete early-pregnancy BMI data, the observed associations cannot be attributed specifically to pre-pregnancy obesity, and our cross-sectional design cannot capture longitudinal changes. Third, cellular heterogeneity is a major limitation of our bulk RNA-seq approach. Although composition estimations suggested that gross shifts did not entirely drive the results, bulk profiling cannot definitively distinguish cell-intrinsic transcriptional regulation from changes in cellular proportions or activation states; matched single-cell data are required to resolve this. Fourth, no plasma cytokine associations remained significant after multiple-testing correction, emphasizing that these findings are strictly hypothesis-generating. Finally, limited availability of maternal and cord blood samples precluded additional ELISA validation of plasma analytes and qRT-PCR validation in PBMCs or CBMCs. The qRT-PCR analysis was therefore limited to available villous placental tissue and should be interpreted as supportive, exploratory validation of selected placental RNA-seq findings. Future studies incorporating larger cohorts, single-cell transcriptomics, and functional validation are needed to establish causality.

In conclusion, this exploratory multi-compartment study suggests that increasing third-trimester BMI is associated with coordinated but compartment-specific molecular signatures across maternal immune cells, placental tissue, and fetal cord blood. Rather than supporting a uniform inflammatory model, our results suggest that maternal metabolic status is linked to distinct immune-metabolic, mitochondrial, vascular-interface, extracellular matrix, and placental endocrine-associated adaptations across the maternal–placental–fetal axis. These candidate pathways provide a framework for validation in larger independent cohorts and for future studies of placental function and fetal immune development.

## Data Availability

The RNA-seq data generated in this study have been deposited in the Gene Expression Omnibus (GEO) under accession number GSE334075. Processed results supporting the conclusions of this article are provided in the Supplementary Material.
