# Supplementary material for: Maternal late-pregnancy BMI is associated with compartment-specific immune-metabolic signatures across the maternal–placental–fetal axis
Source: Front Cell Dev Biol. 2026 Oct 28;14:1899628. doi: 10.3389/fcell.2026.1899628 (PMC13752171; doi:10.3389/fcell.2026.1899628)
Supplement: Supplementary file 1 [file Table1.docx]

# 1. Supplementary Table S1. Clinical and demographic characteristics of the Luminex plasma cohort.

| **Characteristic** | **Luminex plasma cohort (N = 34)^1^** |
| --- | --- |
| **Race** |  |
| Black or African American | 34 (100.0%) |
| **Ethnicity** |  |
| Hispanic | 1 (2.9%) |
| Non-Hispanic | 33 (97.1%) |
| **Maternal BMI, kg/m²** | 33.2 (29.4, 40.1) |
| **BMI tertile** |  |
| Low | 12 (35.3%) |
| Mid | 11 (32.4%) |
| High | 11 (32.4%) |
| **Systolic blood pressure, mmHg** | 121.5 (112.0, 130.0) |
| **Diastolic blood pressure, mmHg** | 75.0 (69.0, 81.0) |
| **Blood glucose, mg/dL** | 82.0 (71.0, 99.0) |
| Missing | 7 |
| **Gestational age at delivery, weeks** | 39.0 (38.0, 39.0) |
| **Infant sex** |  |
| Female | 17 (51.5%) |
| Male | 16 (48.5%) |
| Missing | 1 |
| **Birth weight, g** | 3,158 (2,950, 3,420) |
| **Birth length, inches** | 19.5 (19.3, 20.1) |
| **Maternal plasma Luminex available** | 31 (91.2%) |
| **Cord plasma Luminex available** | 31 (91.2%) |
| **Paired maternal and cord plasma available** | 28 (82.4%) |
| ^1^n (%); Median (Q1, Q3) | |
| Values are shown as median (Q1, Q3) for continuous variables and n (%) for categorical variables. | |
| Maternal BMI refers to BMI recorded during the third trimester of pregnancy. | |
| The Luminex plasma cohort included all participants with available maternal and/or cord plasma Luminex data used in the exploratory plasma analyses. | |
